# 25309 Applying Community Health Priorities to the Translational Research Agenda

**DOI:** 10.1017/cts.2021.579

**Published:** 2021-03-30

**Authors:** Jacquelyn Fede, Stephen Kogut, Anthony Hayward, John F. Stevenson, Amy Nunn, Julie Plaut, Judy A. Kimberly

**Affiliations:** 1 University of Rhode Island; 2 Brown University

## Abstract

ABSTRACT IMPACT: This work has begun to provide the foundation for better ensuring that translational research funded and supported by our IDeA-CTR grant is more directly addressing community- and stakeholder-authored health priorities. OBJECTIVES/GOALS: In order to effectively engage diverse, societal perspectives, we aimed to determine the relevance and feasibility of purposefully aligning translational research with health priorities adopted by the RI Department of Health, health-focused organizations, and community leaders. METHODS/STUDY POPULATION: Individuals from 27 community organizations in RI were asked, ‘What are your health related goals for your community’ and submitted responses online for 2 weeks. Participants generated 71 goals which they sorted into meaningful clusters and rated for importance and feasibility. Clusters were contrasted with RI health priorities to gauge alignment and saturation. In the next phase of this project, researchers and service users funded by Advance-CTR will be asked in routinely administered surveys how their current work may align with RI health goals and whether their future work can feasibly be connected to those priorities. RESULTS/ANTICIPATED RESULTS: Using Group Concept Mapping software, the 71 health goals identified by community organization representatives were fit into an 8-cluster model. Results suggested highest importance placed on Accessible & Healthy Housing (M=4.12, SD=0.29), Community (M=4.08, SD=0.28), Youth (M=4.04, SD=0.49) and Mental Health (M=4.03, SD=0.46). State agency priorities were found to overlap substantially with clusters defined by community leaders. We expect researchers will rate clusters differently, and find some community-endorsed health goals more relevant to their work than others. Perceived feasibility of tailoring future research to state health goals is expected to vary widely by item and researcher. DISCUSSION/SIGNIFICANCE OF FINDINGS: We intend to: 1) facilitate discussions about successes and challenges of translating community-authored priorities into research, and 2) foster better understanding between researchers and the communities they aim to serve on the role of CTR for addressing health challenges in the state.

